# Structural mechanisms of human RecQ helicases WRN and BLM

**DOI:** 10.3389/fgene.2014.00366

**Published:** 2014-10-29

**Authors:** Ken Kitano

**Affiliations:** Graduate School of Biological Sciences, Nara Institute of Science and TechnologyIkoma, Japan

**Keywords:** Werner syndrome, Bloom syndrome, WRN, BLM, DNA helicase, Holliday junction, structural biology

## Abstract

The RecQ family DNA helicases Werner syndrome protein (WRN) and Bloom syndrome protein (BLM) play a key role in protecting the genome against deleterious changes. In humans, mutations in these proteins lead to rare genetic diseases associated with cancer predisposition and accelerated aging. WRN and BLM are distinguished from other helicases by possessing signature tandem domains toward the C terminus, referred to as the RecQ C-terminal (RQC) and helicase-and-ribonuclease D-C-terminal (HRDC) domains. Although the precise function of the HRDC domain remains unclear, the previous crystal structure of a WRN RQC-DNA complex visualized a central role for the RQC domain in recognizing, binding and unwinding DNA at branch points. In particular, a prominent hairpin structure (the β-wing) within the RQC winged-helix motif acts as a scalpel to induce the unpairing of a Watson–Crick base pair at the DNA duplex terminus. A similar RQC-DNA interaction was also observed in the recent crystal structure of a BLM-DNA complex. I review the latest structures of WRN and BLM, and then provide a docking simulation of BLM with a Holliday junction. The model offers an explanation for the efficient branch migration activity of the RecQ family toward recombination and repair intermediates.

## INTRODUCTION

RecQ helicases, a family of DNA unwinding enzymes that belong to the SF2 superfamily helicases, play crucial roles at multiple steps in DNA recombination, replication and repair. Whereas the genomes of bacteria typically encode a single recQ gene, the human genome contains five recQ genes that encode Werner syndrome protein (WRN), Bloom syndrome protein (BLM), RECQ1, RECQ4, and RECQ5. Mutations in WRN and BLM are associated with the rare genetic diseases Werner and Bloom syndromes, respectively. These two diseases are characterized by a high frequency of cancer predisposition, illustrating the primary importance of WRN and BLM in preventing tumorigenesis. Indeed, cells derived from aﬄicted patients show pronounced genomic instabilities such as sister chromatid exchange and telomere shortening.

The Werner and Bloom syndromes, however, are also characterized by many distinct clinical symptoms: Werner patients display features of accelerated aging including the early onset of osteoporosis, atherosclerosis, arteriosclerosis, type II diabetes and cataracts (Goto, 2000; Goto et al., 2013; Oshima et al., 2013), while Bloom patients display severe growth retardation with short stature, immunodeficiency, sunlight sensitivity and a predisposition to a wide spectrum of cancers (Manthei and Keck, 2013). The different clinical features of the disorders, and the fact that the functional loss of either WRN or BLM cannot be compensated for by the presence of the other protein (or of other RecQ members), support the notion that WRN and BLM have distinct functions in cells.

To date, a number of reviews on the biological functions of WRN and BLM have been published, including the latest ones that discuss the diverse genome-maintenance mechanisms of the RecQ family (Larsen and Hickson, 2013; Croteau et al., 2014). In this review, I will focus on structural aspects of WRN and BLM, which are an exciting area of current RecQ research. In particular, structures and functions of the RecQ C-terminal (RQC) and helicase-and-ribonuclease D-C-terminal (HRDC) domains of WRN and BLM are discussed. These two domains are conserved in tandem on the C-terminal side of each protein’s ATPase domain, but also display several divergent features; the sequence identity within the ATPase domain of WRN and BLM is ∼30%, while the identities within the RQC and HRDC domains are ∼10 and ∼20%, respectively. An understanding of the structural and functional differences between these domains may yield insights into the onset of the two distinct diseases.

Furthermore, I present a novel docking simulation of BLM with a Holliday junction (HJ), using the recently determined crystal structure of a BLM-DNA complex (Swan et al., 2014). The model offers explanations for the efficient branch migration activities of BLM and also of WRN.

## DOMAINS OF WRN AND BLM

**FIGURE 1:**
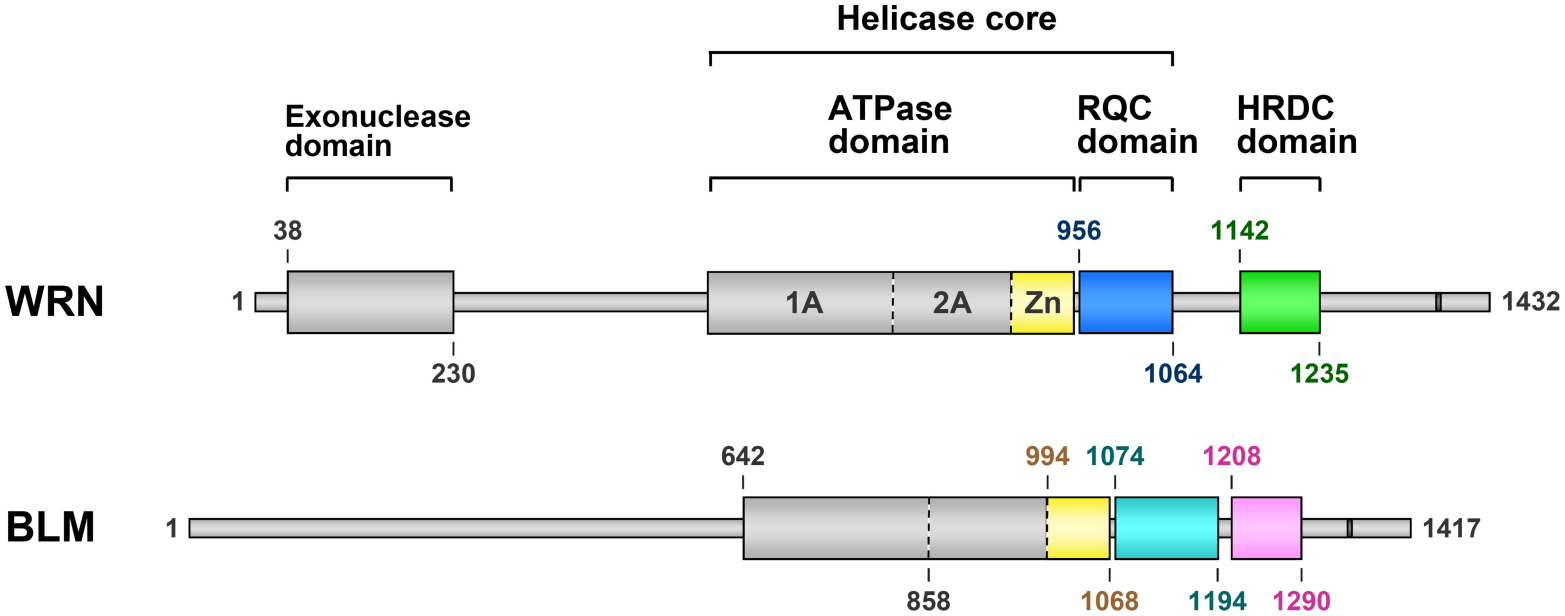
**Domain diagrams of human WRN and BLM.** Werner syndrome protein and Bloom syndrome protein share three structurally folded domains comprising an ATPase domain, an RQC domain (colored blue in WRN and cyan in BLM) and an HRDC domain (green and pink, respectively). The Zn subdomain (yellow) is located at the C-terminal end of the ATPase domain. Nuclear localization signals of WRN (Matsumoto et al., 1997) and BLM (Kaneko et al., 1997) are depicted as dark gray bars. The domain boundaries (a.a. numbers) were determined from the available 3D structures of WRN (Hu et al., 2005; Perry et al., 2006; Kitano et al., 2007, 2010) and BLM (Kim and Choi, 2010; Sato et al., 2010; Kim et al., 2013; Swan et al., 2014).

Domain diagrams of WRN and BLM are shown in **Figure 1**. WRN and BLM are multi-domain helicases composed of 1,432 and 1,417 amino acids (a.a.), respectively. The two proteins share the structured ATPase, RQC, and HRDC domains, while an exonuclease domain is present only at the N terminus of WRN. Previous crystal structures of the exonuclease domain from human (Perry et al., 2006) and mouse (Choi et al., 2007) WRNs in the absence of DNA suggested a nuclease mechanism mediated by two metal ions, although the *in vivo* role of this domain is still unknown.

## RQC DOMAIN

### WRN RQC

The RQC domain, which is tethered to the zinc-binding subdomain (Zn) of the ATPase domain with a short linker, is unique to the RecQ family of proteins. This region folds into a winged-helix motif, a subset of the helix-turn-helix superfamily (Hu et al., 2005; Kitano et al., 2010; Kim et al., 2013; Swan et al., 2014). Helix-turn-helix motifs including the winged helix are known as major double-stranded (ds) DNA-binding domains and are found in many nuclear proteins (Gajiwala and Burley, 2000; Harami et al., 2013).

**FIGURE 2:**
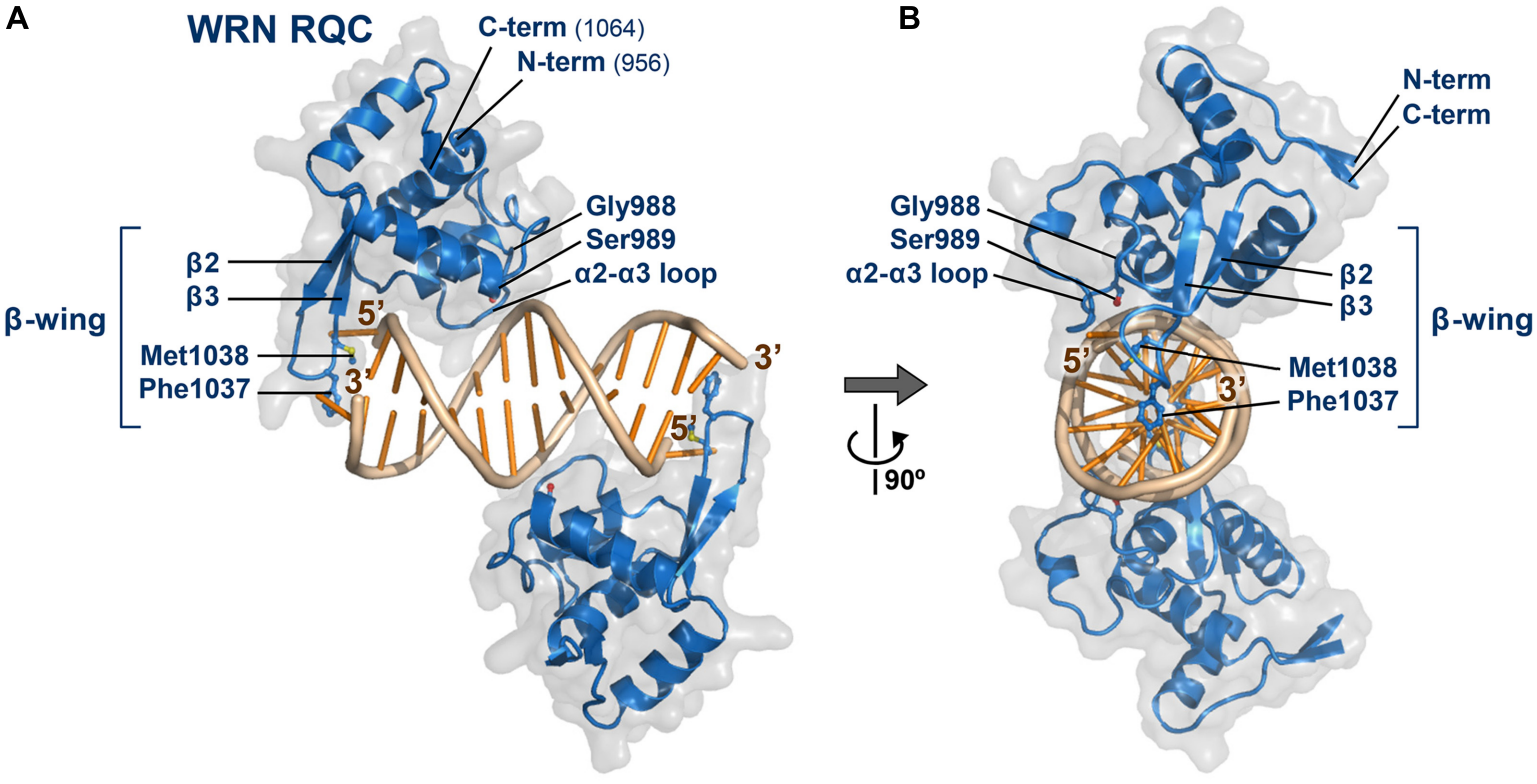
**Structure of the WRN RQC domain bound to dsDNA. (A)** Crystal structure of WRN RQC bound to the 14-base-pair duplex (PDB ID: 3AAF; Kitano et al., 2010). The two RQC monomers (blue) bind to each DNA blunt terminus and unpair the terminal base pairs. The molecular surfaces of each domain are shown in transparent gray. Secondary-structure elements are labeled, and side chains of the key interacting amino acids are shown as stick models. The unpaired 5′-nucleotide is held tightly by RQC to prevent its reannealing, whereas the 3′-nucleotide is mostly disordered. All figures displaying 3D structures within this paper were prepared using PyMOL (DeLano Scientific). **(B)** View following 90° rotation along the y-axis.

**Figure 2** shows the co-crystal structure of the WRN RQC domain bound to a DNA duplex (Kitano et al., 2010), whose determination in 2010 represented the first example of a RecQ-DNA complex. The structure revealed two unexpected features of the RQC domain. First, the RQC domain binds duplex DNA in a novel DNA-interaction mode that differs from all known examples of winged-helix and other helix-turn-helix proteins. The recognition helix, a principal component of helix-turn-helix motifs that are usually embedded within DNA grooves, was unprecedentedly excluded from the interaction. Second, the structure successfully captured a DNA-unwinding event by the RQC domain. The RQC domain specifically interacted with a blunt end of the DNA duplex and, in the absence of any other domain, unpaired a Watson–Crick base pair using the prominent hairpin structure β2–β3, which corresponds to the so-called β-wing of the winged-helix fold.

### BLM RQC

**FIGURE 3:**
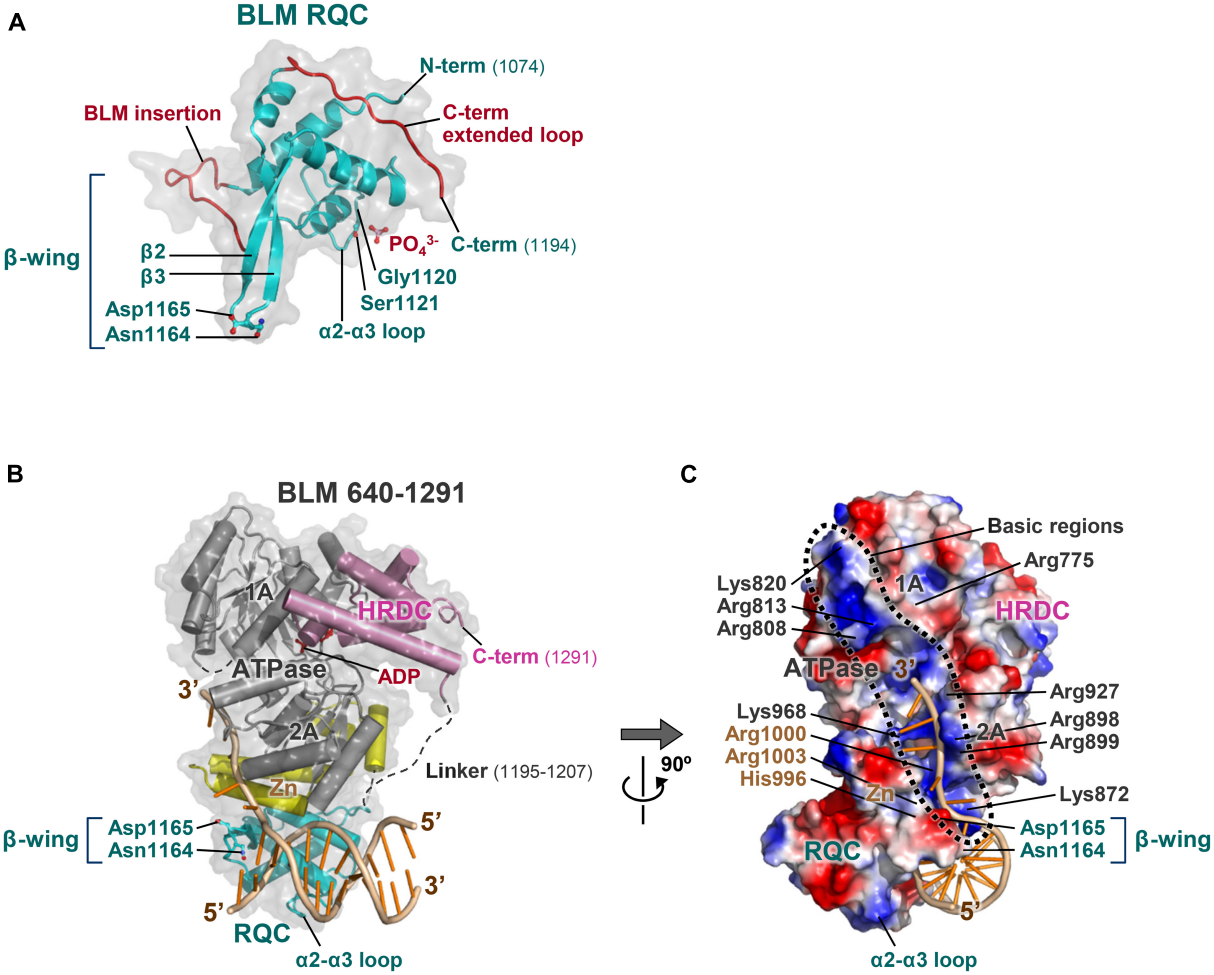
**Structures of the BLM RQC domain and of BLM 640–1291 bound to DNA. (A)** Crystal structure of BLM RQC bound to a phosphate ion (PDB ID: 3WE2; Kim et al., 2013). Two BLM-specific loop regions, BLM-insertion (a.a. 1093–1106) and C-term extended loop (a.a. 1183–1194), are colored red. The phosphate ion forms a hydrogen bond with Ser1121, mimicking one of the phosphate groups in DNA substrates. **(B)** Crystal structure of BLM 640–1291 bound to a 3′-overhang duplex (PDB ID: 4O3M; Swan et al., 2014). The RQC and HRDC domains are in cyan and pink, respectively, while the Zn subdomain within the ATPase domain is in yellow. ADP (red) is bound to the inter-subdomain cleft between the ATPase subdomains 1A and 2A (gray). **(C)** Surface potential representation of BLM 640–1291 in a view following 90° rotation along the y-axis. The basic regions along the ATPase domain are encircled by a dashed line, and include a number of basic residues: Arg775, Arg808, Arg813, and Lys820 within subdomain 1A; Lys872, Arg898, Arg899, Arg927, and Lys968 within subdomain 2A; and His996, Arg1000, and Arg1003 within the Zn subdomain.

Last year, the crystal structure of the BLM RQC domain bound to a phosphate ion (**Figure 3A**) was determined (Kim et al., 2013), and, subsequently, the co-crystal structure of a BLM large fragment (a.a. 640 –1291) in complex with a 3′-overhang DNA duplex (**Figures 3B,C**) was determined (Swan et al., 2014). The latter structure includes all of the ATPase, RQC, and HRDC domains, but interactions with the duplex region of the DNA were concentrated on the RQC domain surface; the BLM RQC domain binds to the dsDNA terminus in the same binding mode as had been observed with the truncated WRN RQC domain (Kitano et al., 2010).

The structure of the BLM RQC domain, however, includes three distinct features (**Figure 3A**). First, aromatic and non-polar residues at the tip of the β-wing, key elements that WRN uses for DNA strand separation, are each replaced by polar and acidic residues in BLM. A detailed discussion of this feature is given below. Second, a BLM-specific 14-a.a. insertion (referred to as the BLM insertion) between the N-terminal helices exhibits a looping-out structure that extends at right angles to the β-wing. Third, the C-terminal residues of BLM RQC adopt a novel extended structure (referred to as the C-term extended loop) by being tightly packed against the domain core. These unique structures in BLM RQC may be associated with the preferential activity of BLM toward HJs (Kim et al., 2013).

### RQC IS AN UNCONVENTIONAL WINGED-HELIX DOMAIN

**FIGURE 4:**
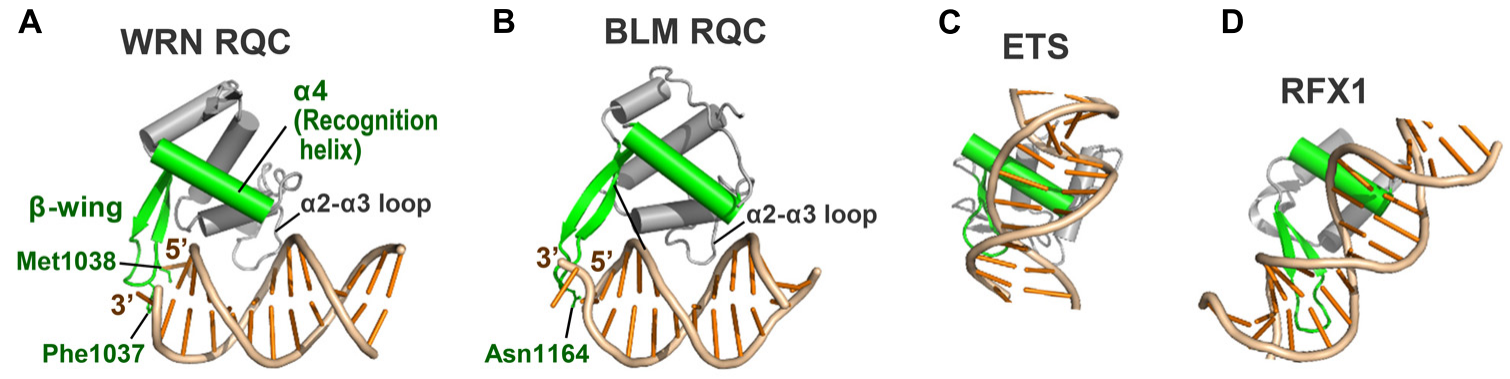
**Comparison of the RQC domains with conventional winged-helix domains. (A)** WRN RQC domain (PDB ID: 3AAF; Kitano et al., 2010). The RQC domain binds the DNA duplex terminus via the α2–α3 loop and the β-wing (colored green), while the helix α4 (recognition helix; also green) is located apart from the DNA. **(B)** BLM RQC domain (PDB ID: 4O3M; Swan et al., 2014). The orientation and colors of the domain are comparable to those in **(A)**. **(C)** PU.1 ETS winged-helix domain (PDB ID: 1PUE; Kodandapani et al., 1996). The recognition helix and β-wing bind the major and minor grooves, respectively. **(D)** RFX1 winged-helix domain (PDB ID: 1DP7; Gajiwala et al., 2000). The recognition helix and β-wing bind the minor and major grooves, respectively.

**Figure 4** shows a comparison of the DNA-binding modes of the WRN (A) and BLM (B) RQC domains with those of the conventional winged-helix domains of the transcription factors ETS (Kodandapani et al., 1996; C) and RFX1 (Gajiwala et al., 2000; D). The conventional winged-helix domains all bind to DNA via principal contacts of a recognition helix (colored green in C, D) deep in the major or minor groove of DNA. This arrangement facilitates sequence-specific DNA binding that can induce a bend in the DNA (Gajiwala and Burley, 2000; Harami et al., 2013).

In contrast, the recognition helix (α4) of WRN RQC (A) and BLM RQC (B) is located more than 4 Å away from the bound DNA and is not involved in the direct interaction with DNA. Instead, the positively charged loop between helices α2 and α3 (the α2–α3 loop) serves as the prominent DNA binding site by interfacing with the major groove of the DNA, and the β-wing (also green) exhibits a unique interaction with the terminus of the duplex. Considering that the fundamental role of the recognition helix is to promote sequence-specific DNA recognition, its exceptional lack of use in RQC seems essential for realizing sequence-independent helicase reactions (Kitano et al., 2010). The RQC domain, unlike the conventional winged-helix domains, does not form a hydrogen bond with the bases or induce a bend in the duplex.

The protruding β-wing within the RQC domain is also essential for WRN and BLM to prevent non-specific binding to DNA, since the β-wing exhibits steric hindrance with linear paired bases (Kitano et al., 2010). Due to this conflict effect, the proteins can bind only to branched sites that contain a terminus of the duplex, a structural explanation for the DNA structure-specific activities of the RecQ family. Electron microscopic analyses of full-length WRN (Compton et al., 2008) and BLM (Huber et al., 2006) also showed that the two proteins do not bind DNA in the interior of the linear B-form conformation.

The α2–α3 loop of the RQC domains plays a major role in the interaction with DNA. On this loop, a conserved serine of WRN (Ser989 in **Figure 2**) and BLM (Ser1121 in **Figure 3A**; the phosphate ion mimics one of the DNA phosphates) forms a hydrogen bond with a backbone phosphate of the DNAs (Kitano et al., 2010; Kim et al., 2013; Swan et al., 2014). Single mutation of these serines disturbs the DNA-binding ability of WRN RQC (Kitano et al., 2010) and BLM RQC (Kim et al., 2013), showing their common importance for DNA interaction. Another key residue on this loop is a glycine (WRN Gly988 and BLM Gly1120), which is adjacent to the serine and important to provide the α2–α3 loop with the flexibility required for DNA interaction (Kitano et al., 2010). Single mutation of Gly1120 in BLM also causes partial loss-of-function of the full-length protein (Mirzaei and Schmidt, 2012).

### THE β-WING, A HAIRPIN SCALPEL FOR DNA STRAND SEPARATION

**FIGURE 5:**
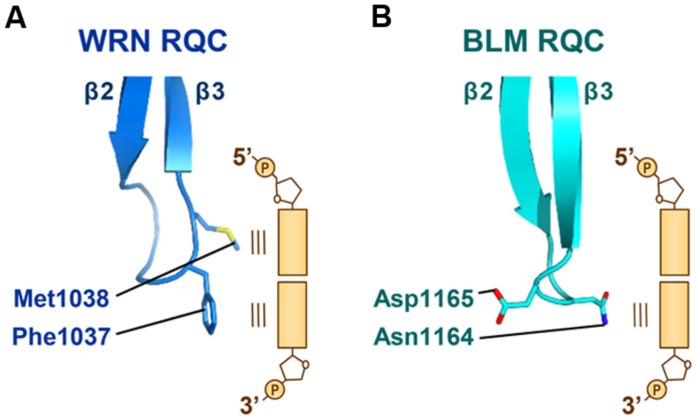
**Interactions of the WRN and BLM β-wings with DNA. (A)** The β-wing of WRN RQC (PDB ID: 3AAF; Kitano et al., 2010) is capped by aromatic (Phe1037) and non-polar (Met1038) residues, which stack onto the last paired bases at the 3′ and 5′ termini, respectively. **(B)** The β-wing of BLM RQC (PDB ID: 4O3M; Swan et al., 2014) is capped by polar (Asn1164) and acidic (Asp1165) residues. Asn1164 interacts with the last paired base at the 3′ terminus, while Asp1165 faces in the other direction.

The β-wing of the RQC domain extends from the edge of the domain surface and, during the helicase catalytic reactions, acts as a scalpel for splitting a DNA duplex. **Figure 5A** shows a schematic depiction of the WRN β-wing interaction with the last paired bases of the partially unwound DNA duplex (Kitano et al., 2010). The aromatic (Phe1037) and non-polar (Met1038) residues at the hairpin tip cut into the stacked bases from the duplex terminus, resulting in a loss of base–base stacking and the separation of both strands.

On the other hand, the β-wing of BLM (**Figure 5B**) is capped by polar (Asn1164) and acidic (Asp1165) residues (Kim et al., 2013; Swan et al., 2014). Asn1164 (the counterpart of WRN Phe1037) also functions to wedge apart the DNA strands by interacting with the last paired base at the 3′ terminus. The importance of both WRN Phe1037 and BLM Asn1164 in DNA-unwinding reactions was confirmed by mutagenesis helicase assays (Tadokoro et al., 2012; Swan et al., 2014). In contrast, the acidic side chain of BLM Asp1165 (the counterpart of WRN Met1038) does not interact with the duplex but faces in the other direction. When binding to multi-stranded DNAs like a HJ, such electronegativity at the tip of the β-wing may result in an electrostatic repulsion against the neighboring DNA strands. In agreement with this idea, the DNA-binding activity of purified BLM is weaker than that of WRN (Kamath-Loeb et al., 2012; Kim et al., 2013). The electrostatic repulsive power between the acidic BLM β-wing and DNA strands, as discussed below, may be adapted for use in catalyzing the branch migration of HJs.

## HRDC DOMAIN

### WRN HRDC AND BLM HRDC

In contrast to the accumulated knowledge pertaining to the unique functions of the RQC domain, the function of the C-terminal HRDC domain remains unclear. In human RecQs, only WRN and BLM possess the HRDC domain, whereas the other three members (RECQ1, RECQ4, and RECQ5) completely lack HRDC sequences (Larsen and Hickson, 2013; Croteau et al., 2014). The existing data concerning WRN (Lee et al., 2005) and BLM (Wu et al., 2005) suggest that the HRDC domain is not essential for conventional helicase activity on forked duplexes.

**FIGURE 6:**
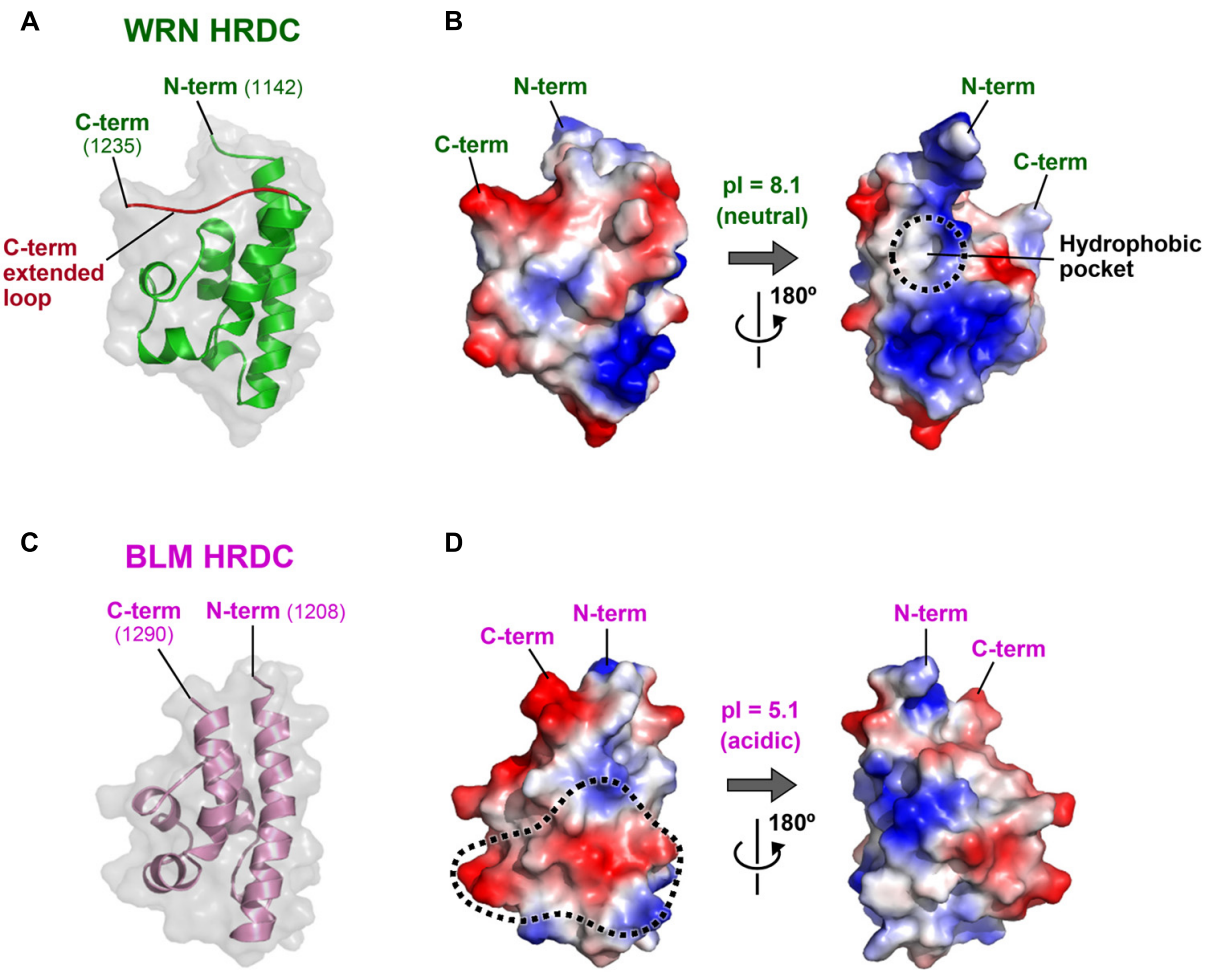
**Structures of the WRN and BLM HRDC domains. (A)** Crystal structure of WRN HRDC (PDB ID: 2E1E; Kitano et al., 2007). The WRN-specific C-term extended loop (a.a. 1227–1235) is colored red. **(B)** Surface potential of WRN HRDC. Front (left) and back (right) views. A WRN-specific hydrophobic pocket at the back surface is encircled by a dashed line. **(C)** NMR structure of BLM HRDC (PDB ID: 2RRD; Sato et al., 2010). The molecule is viewed in the same orientation as in **(A)**. **(D)** Surface potential of BLM HRDC. A front (left) surface area corresponding to the proposed DNA interaction area of Sgs1 (Liu et al., 1999) is encircled by a dashed line.

**Figure 6** shows the crystal structure of the WRN HRDC domain (A, B; Kitano et al., 2007) and the NMR structure of the BLM HRDC domain (C, D; Kim and Choi, 2010; Sato et al., 2010). The two HRDC domains fold into a common globular bundle of five α-helices and one 3_10_-helix connected by short loop regions. However, the amino acids located on the domain surfaces are poorly conserved, yielding distinct surface properties for each protein. For example, the WRN HRDC domain surface retains both acidic and basic regions (B), whereas the BLM HRDC surface is largely electronegative with many acidic residues exposed to the solvent (D). The isoelectric point (pI) of BLM HRDC (5.1) is also much lower than that of WRN HRDC (8.1; Sato et al., 2010).

The distinct charge distributions of the two HRDC domains suggest different roles for the domain in each protein. As its name implies, the HRDC domain was originally found in several bacterial DNA helicases such as PcrA (Subramanya et al., 1996; Velankar et al., 1999) and Rep (Korolev et al., 1997), in addition to the RNase D family of nucleases (Zuo et al., 2005). Consequently, interest in the HRDC domains has focused on their DNA-binding ability (Morozov et al., 1997). The isolated HRDC domain of Sgs1 (the yeast ortholog of BLM) was shown to bind DNA weakly using the electropositive surface area of the domain (Liu et al., 1999). However, both the WRN and BLM HRDC structures show that the proposed DNA-binding surface area of Sgs1 is not conserved (Kitano et al., 2007; Sato et al., 2010). The corresponding region of BLM HRDC (encircled by a dashed line in **Figure 6D**) is highly electronegative, and is therefore unlikely to be involved in direct DNA interaction. Consistent with these observations, neither of the purified WRN (Kitano et al., 2007) nor BLM HRDCs (Sato et al., 2010; Kim et al., 2013) exhibit detectable DNA-binding ability *in vitro*.

### POSSIBLE FUNCTION OF WRN HRDC

As depicted in **Figure 1**, the HRDC domains of WRN and BLM are connected to their adjacent RQC domains by different lengths of linker. The linker of WRN comprises 77 residues (a.a. 1065–1141), which is six times longer than that of BLM (13 residues; a.a. 1195–1207). The long linker region of WRN is probably unstructured (Kitano et al., 2007), which may contribute to the spatial separation of the HRDC domain from the RQC domain. Such isolation may be advantageous for interactions with other proteins. For example, the WRN HRDC domain may interact with other protein partner(s) that recognize DNA double-strand breaks (DSBs), thus facilitating indirect recruitment of WRN to the site for repair (Lan et al., 2005; Kitano et al., 2007).

The WRN HRDC domain also possesses a C-term extended loop (colored red in **Figure 6A**) that is tightly packed against the upper surface of the folding core. This extra structure increases the surface area of WRN HRDC, and assists in the formation of WRN-specific structures such as a hydrophobic pocket at the back of the domain (encircled by a dashed line in **Figure 6B**). This hydrophobic pocket is a candidate for interaction with other proteins (Kitano et al., 2007).

## ATPase DOMAIN

The ATPase domain is the largest and most highly conserved component of the RecQ family (**Figure 1**). The domain is constituted of two RecA-like subdomains (1A and 2A) that are characteristic of a wide variety of DNA and RNA helicases (Singleton et al., 2007). The ATPase domain is responsible for binding and hydrolysis of ATP (Vindigni et al., 2010; Swan et al., 2014). The C-terminal portion of the ATPase domain also includes the Zn subdomain (colored yellow), which is unique to the RecQ family and is often combined with the RQC domain sequence. Crystal structures of bacterial (Bernstein et al., 2003) and human (Pike et al., 2009; Swan et al., 2014) RecQs showed that the Zn subdomain is tightly packed against subdomain 2A of the ATPase domain, and is thus structurally a part of the ATPase domain.

By analogy with other helicases (Singleton et al., 2007), the ATPase domain of the RecQ family was originally referred to as a “helicase domain.” However, this designation often caused misunderstanding, since the domain itself does not possess helicase activities; the isolated ATPase domains of WRN (Von Kobbe et al., 2003; Lee et al., 2005) and BLM (Janscak et al., 2003) do not exhibit efficient DNA-binding or unwinding activity. As described above, the RQC domain, not the ATPase domain, constitutes the primary DNA-binding site in members of the RecQ family and also catalyzes the direct unpairing of DNA duplexes (Kitano et al., 2010; Swan et al., 2014).

The ATPase domains of WRN and BLM, therefore, function simply as an ATP-dependent DNA translocation module that supplies a driving force for the helicase reactions. The ATPase and RQC domains are both required for the processive helicase reactions, combining to form a “helicase core” in the RecQ family.

## BLM-HJ BINDING MODEL

### DISSOLUTION OF DOUBLE HJ BY BLM AND TOPOISOMERASE IIIα

Unlike most other helicases, WRN and BLM (and some other RecQ members) preferentially act on DNA structures that resemble recombination and repair intermediates (Larsen and Hickson, 2013; Croteau et al., 2014). Such structure-specific activities of RecQs account for many of their key functions in DNA metabolic pathways. For example, BLM acts in concert with topoisomerase IIIα to resolve the DSB repair intermediate double HJ, producing exclusively non-crossover products (Wu and Hickson, 2003; Wu et al., 2005; Plank et al., 2006; Seki et al., 2006). This process, referred to as double HJ dissolution, is crucial for suppressing the sister-chromatid exchanges that cause early neoplastic transformation of cells (Larsen and Hickson, 2013; Manthei and Keck, 2013). In double HJ dissolution, a branch migration activity of BLM (Karow et al., 2000) is used to efficiently bring two HJs toward each other, until they form a hemi-catenane intermediate that can be decatenated by topoisomerase IIIα.

**FIGURE 7:**
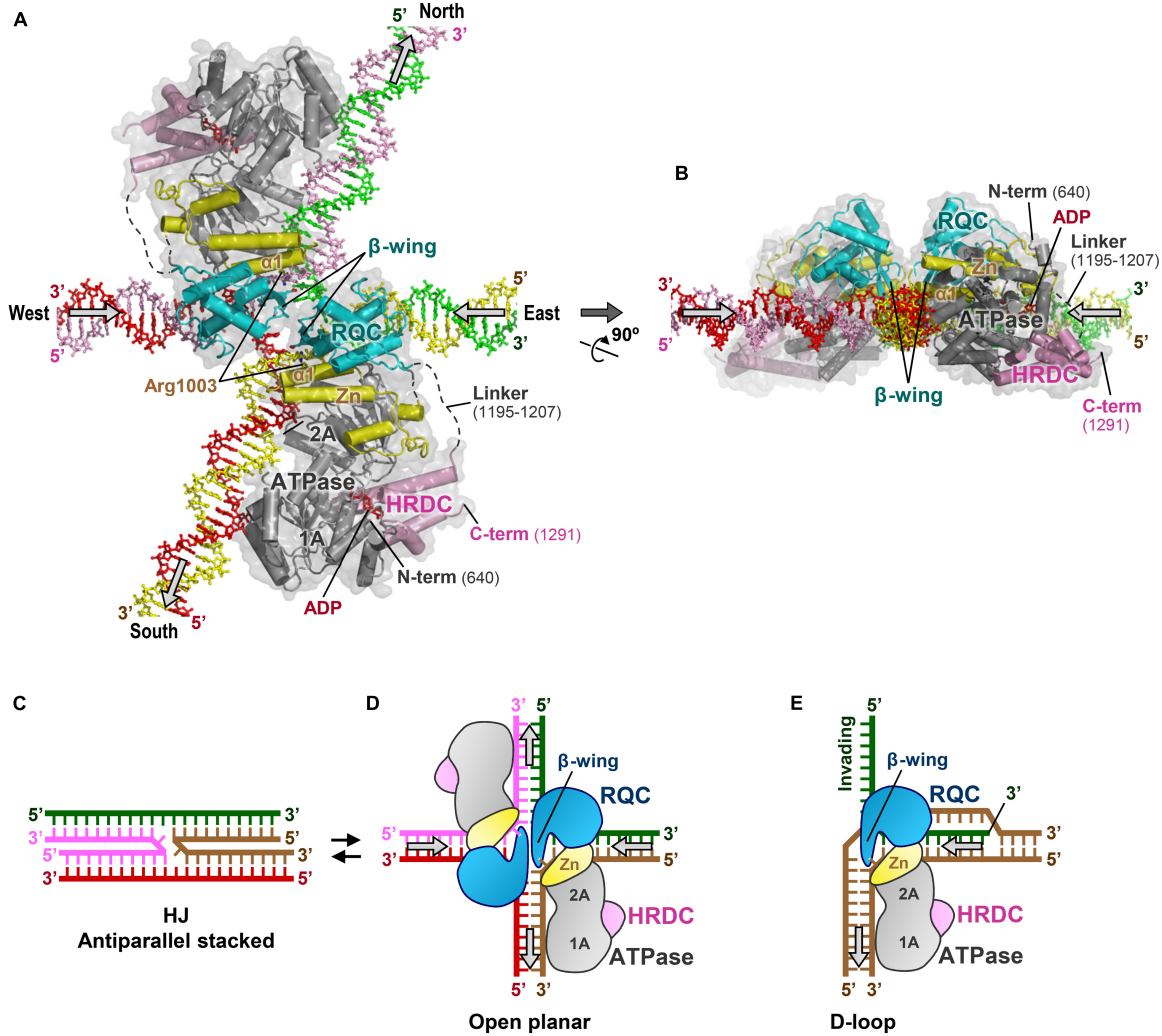
**Binding model of BLMs to HJ. (A)** 3D docking simulation of a BLM dimer to a HJ. BLMs are in the same colors as in **Figure 3B**. The arrows on the HJ arms represent the directions of DNA translocations required for branch migration. This model was constructed in silico by the following procedures: first, the structure of BLM 640–1291 (PDB ID: 4O3M; Swan et al., 2014) was superimposed onto our previous binding model of the WRN RQC-HJ complex (Kitano et al., 2010) using the RQC domain and DNA duplex region as references. The north–south arms of the HJ were then manually tilted by 21° on the same plane using Coot (Emsley et al., 2010), so that the ATPase subdomains 1A and 2A can simultaneously interact with the duplexes without steric conflicts. The present interactions between the RQC domains and the HJ are consistent with a previous mutagenesis binding assay of BLM RQC with HJ (Kim et al., 2013). **(B)** View after 90° rotation along the x-axis. **(C)** Schematic representation of a HJ in the antiparallel stacked conformation. **(D)** Schematic representation of **(A)**, with the HJ in the ideal open-planar conformation. **(E)** Schematic representation depicting release of the 3′-invading strand (green) by WRN or BLM (Kitano et al., 2010).

In **Figures 7A,B** I present a new binding model of a BLM dimer with a HJ. This model has been constructed in silico by superimposing the BLM 640–1291 structure (Swan et al., 2014) onto our previous docking simulation of the WRN RQC-HJ complex (Kitano et al., 2010). The BLM 640–1291 structure was used without modification, while the north and south arms of the HJ (the vertical duplexes in **Figure 7A**) were manually tilted by 21° rotation from their ideal vertical positions, to avoid steric conflicts with the two ATPase domains. The obtained model shows that the two molecules of BLM can simultaneously bind to the single HJ with no significant steric hindrance to each other. Each RQC domain binds the east or west (the horizontal) arm of the HJ with twofold symmetry, while the ATPase domains interact with the north and south arms. Binding of a BLM dimer to a single HJ is also suggested by a recent single-molecule visualization study (Gyimesi et al., 2013).

In the electrostatic surface potentials of BLM 640–1291 (**Figure 3C**), a line of basic regions (encircled by a dashed line) that traverses subdomains 1A, 2A, and Zn of the ATPase domain is observed. The lower part of these regions binds to the 3′-overhang ssDNA, suggesting a role for these regions as a DNA translocation route for the helicase reactions. In the present BLM-HJ model (**Figure 7A**), the basic regions in each ATPase domain run precisely in parallel with the north or south duplexes, representing the best operational arrangement for the translocations of the two arms.

### PROPOSED BRANCH MIGRATION MECHANISM

The BLM-HJ model (**Figures 7A,B**) offers several plausible explanations for the branch migration reactions undertaken by BLM. First, co-insertion of the two acidic β-wings into a small hole of the HJ enables the BLMs to catalyze simultaneous unpairing of the east and west arms of the HJ. This is reminiscent of the acidic hairpin of bacterial RuvA, a protein that also catalyzes branch migration of HJs (Ingleston et al., 2000; Yamada et al., 2004). By analogy with the proposed mechanism for RuvA, negative charges on the BLM β-wings may serve to repel the DNA backbones from the junction center and/or to repel the other β-wing within the same hole by electrostatic repulsion. The resultant mechanical force to enlarge the hole could result in the disruption of base pairs near the crossover point, thereby enhancing strand-exchange reactions.

Second, nucleotides that are newly unpaired by the RQC domains may be translocated under guidance of the Zn subdomain, which abuts the ATPase subdomain 2A. In the present model, the first helix, α1 (a.a. 995–1007), of each Zn subdomain is extended in parallel with the yellow or pink strand at the intersection, exposing the basic residue Arg1003 toward the unpaired 3′-nucleotides. This residue probably forms a salt bridge with the DNA backbone phosphate. Therefore, the two Zn subdomains located around the central hole seem likely to act as a guiderail for the unpaired 3′-nucleotides to move smoothly into the north-south duplexes, helping them to rapidly anneal with new partners. At the same time, the Zn subdomain would also act as a joint to adjust the relative orientation of the ATPase domain against the RQC domain.

Third, the two ATPase domains would pull the north and south duplexes in opposite directions, by the conventional inch-worm mechanism (Singleton et al., 2007). Hydrolysis of an ATP at the interface of the ATPase subdomains 1A-2A would result in a rigid-body movement of subdomain 1A, a conformational change of the ATPase domain as a motor. The resultant translocations of the north–south duplexes by the ATPase domains would in turn allow each RQC domain to melt the next base pairs of the east–west duplexes, thereby driving the branch migration reactions.

In summary, although a genuine BLM-HJ co-crystal structure is still lacking, the proposed binding model yields a possible HJ branch migration mechanism for BLM, in which DNA unwinding by the RQC domains and DNA annealing/translocation by the ATPase domains are effectively coordinated.

### SEPARATION OF UNPAIRING MODULE FROM TRANSLOCATION MODULE

**FIGURE 8:**
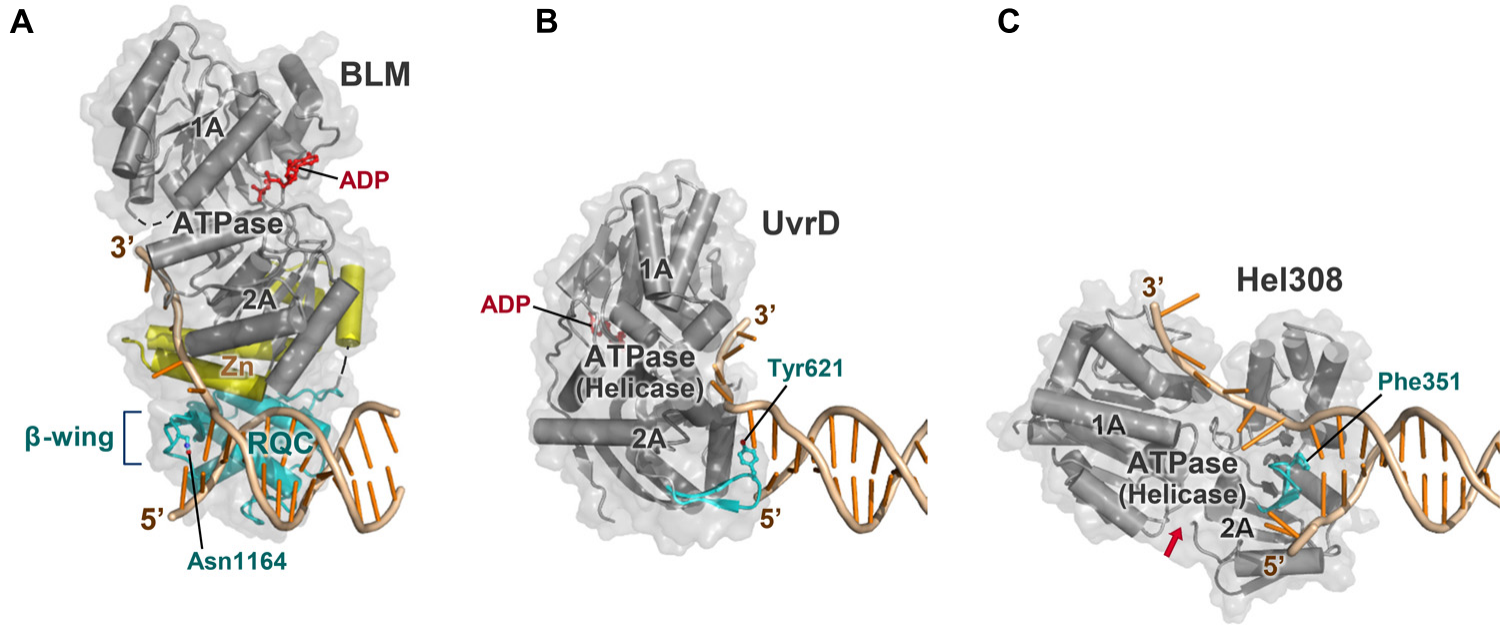
**Comparison of BLM with other helicases. (A)** BLM 640–1291 bound to a 3′-overhang duplex (PDB ID: 4O3M; Swan et al., 2014). The strand-separating element (β-wing) is within the RQC domain (colored cyan), which is bridged to the ATPase domain (gray) by the Zn subdomain (yellow). The complex is viewed in the same orientation as in **Figure 3B** except for the omission of the HRDC structure; in **(A–C)**, other portions of the enzyme outside the helicase core are omitted for clarity. **(B)** ATPase (helicase) domain of *E. coli* UvrD bound to a 3′-overhang duplex (PDB ID: 2IS6; Lee and Yang, 2006). The strand-separating element (β-hairpin; cyan) is included within the ATPase subdomain 2A. The structure is comparable to that of PcrA ( Velankar et al., 1999). **(C)** ATPase (helicase) domain of archaeal Hel308 bound to a 3′-overhang duplex (PDB ID: 2P6R; Buttner et al., 2007). The strand-separating element (β-hairpin) is also within the ATPase subdomain 2A. The ATP-binding cleft is indicated by a red arrow. In **(A–C)**, Asn1164 (BLM), Tyr621 (UvrD), and Phe351 (Hel308), respectively, stack onto the last paired base at the 3′ terminus.

Many other helicases such as bacterial UvrD (Lee and Yang, 2006; **Figure 8B**) and archaeal Hel308 (Buttner et al., 2007; **Figure 8C**) also possess a conserved β-hairpin to act as an unwinding element. However, these hairpins are located directly within the ATPase domains (i.e., helicase domains) and cannot be inserted into the narrow hole of the HJ, since severe steric conflicts would occur between their ATPase domains and DNA strands (Kitano et al., 2010). This is the reason why PcrA (a homolog of UvrD) cannot efficiently promote migration of HJs (Constantinou et al., 2000; Karow et al., 2000).

In contrast, WRN and BLM prepare the unwinding element in the compact RQC domain (**Figure 8A**). For the RecQ family, separation of the unwinding module (the RQC domain) from the translocation module (the ATPase domain) is likely to be crucial to process multi-stranded DNAs such as the HJs.

### POSSIBLE FUNCTION OF BLM HRDC

The co-crystal structure of the BLM-DNA complex (**Figure 3B**) also showed that the BLM HRDC domain is situated ∼28 Å apart from the DNA substrate and binds to the ATPase domain within the same molecule (Swan et al., 2014). The domain-domain interface includes an ATP-binding cleft formed between the ATPase subdomains 1A and 2A. Therefore, the BLM HRDC domain may somehow be associated with the hydrolysis of ATP within the ATPase domain, although the detailed mechanism of action is not known.

On the other hand, the HJ is known to adopt a dynamic structure between antiparallel stacked (**Figure 7C**) and open planar conformations (**Figure 7D**; Liu and West, 2004). The former is favored in the presence of divalent cations such as Mg^2+^, but is inhibitory to branch migration reactions due to its closed structure, stabilized by strong van der Waals contacts and hydrogen bonds (Ortiz-Lombardia et al., 1999; Eichman et al., 2000). Therefore, proteins that promote branch migration must first open the four arms, as does RuvA, which binds exclusively to the open planar conformation of the HJ (Ingleston et al., 2000; Yamada et al., 2004).

In the current BLM-HJ model (**Figures 7A,B**), the two HRDC domains are located in the empty spaces between the north–west and east–south arms of the HJ. Considering the electronegativity of the BLM HRDC domain surface, the HRDC domain may act as a wedge to open the HJ arms by electrostatic repulsion (Kim et al., 2013).

## OTHER DNA SUBSTRATES

In addition to the established importance of BLM in double HJ dissolution, BLM may also be required for other genomic events such as segregation of sister chromatids in mitosis (Chan et al., 2007; Larsen and Hickson, 2013; Manthei and Keck, 2013) as well as the recombination process in meiosis (De Muyt et al., 2012; Zakharyevich et al., 2012). Although the precise *in vivo* substrates of BLM in these pathways remain to be elucidated, a mechanism similar to the branch migration function of BLM in resolving multi-stranded DNAs may be utilized.

WRN can also catalyze branch migration of HJs *in vitro* (Constantinou et al., 2000), but fails to substitute for BLM in double HJ dissolution reactions (Wu et al., 2005). Alternatively, WRN may be involved in another DSB repair pathway such as non-homologous end-joining (Lan et al., 2005; Yan et al., 2005) by interacting with its key protein Ku70/80 (Croteau et al., 2014). Furthermore, WRN also plays a role in protecting chromosome ends by interacting with telomere maintenance proteins like TRF2 and POT1 (Croteau et al., 2014). **Figure 7E** shows a schematic view of the displacement loop (D-loop) bound by WRN (or BLM). Since the structure of the D-loop is comparable to that of the right half of the HJ (Kitano et al., 2010), it is tempting to speculate that WRN catalyzes the dissociation of telomeric D-loops in the replication and recombination processes (Opresko et al., 2004; Brosh, 2013).

WRN and BLM are additionally capable of unwinding non-Watson-Crick/Hoogsteen base pairs such as G-quadruplex (G4) DNA (Larsen and Hickson, 2013; Croteau et al., 2014). The G4-unwinding activity of WRN and BLM may be important for the efficient replication of telomeres (Brosh, 2013) as well as for the regulation of gene expression (Johnson et al., 2010; Nguyen et al., 2014). The purified RQC domain of BLM binds to G4 DNA with high affinity (Huber et al., 2006). Future structural studies of complexes with G4 DNA should reveal the mechanism by which WRN and BLM unwind such abnormal DNA structures.

## CONCLUDING REMARKS

Recent advances in the structural studies of WRN and BLM, in particular the discovery of the “DNA zip-slider” function of the RQC domain to catalyze strand separation, have greatly improved our understanding of WRN and BLM in terms of their preferential activities toward recombination and repair intermediates. In this paper, I have focused on the structures of WRN and BLM, but it should be mentioned that other important RecQ structures that could not be discussed here are also available, including those of *Escherichia coli* RecQ (Bernstein et al., 2003; Bernstein and Keck, 2005), *Deinococcus radiodurans* RecQ (Killoran and Keck, 2008; Liu et al., 2013) and human RECQ1 (a protein that is not associated with genetic disease; Pike et al., 2009). The structure of *E. coli* RecQ without DNA (Bernstein et al., 2003) gave us the first structural image of the RecQ-family helicase core, although recent data imply that the β-wing of bacterial RecQs is not involved in DNA unwinding (Pike et al., 2009; Hoadley and Keck, 2010). The β-wing of RECQ1 (Pike et al., 2009), in contrast, probably functions in a manner similar to that of WRN. Reviews on these structures are available elsewhere (Killoran and Keck, 2006; Vindigni et al., 2010).

Besides their biological importance in the prevention of tumorigenesis and accelerated aging, WRN and BLM are also new targets for cancer chemotherapy (Futami et al., 2007; Opresko et al., 2007; Arai et al., 2011; Moser et al., 2012; Brosh, 2013). Recent high-throughput screens of chemical compound libraries identified two compounds, NSC19630 (Aggarwal et al., 2011) and ML216 (Nguyen et al., 2013), as specific inhibitors of WRN and BLM, respectively. Although the mechanism by which NSC19630 interferes with WRN function is unknown, ML216 was shown to inhibit the helicase activity of BLM 636–1298, a fragment similar to that used in the structure determination (Swan et al., 2014), by competing with its DNA-binding activity (Nguyen et al., 2013). Future co-crystallizations of WRN and BLM with these inhibitors may lead us to novel drug design strategies targeting the RecQ family of proteins.

## Conflict of Interest Statement

The author declares that the research was conducted in the absence of any commercial or financial relationships that could be construed as a potential conflict of interest.
